# Multimodal deep learning for dementia classification using text and audio

**DOI:** 10.1038/s41598-024-64438-1

**Published:** 2024-06-16

**Authors:** Kaiying Lin, Peter Y. Washington

**Affiliations:** 1 Department of Information and Computer Science, University of Hawai’i, Honolulu, 96822 USA; 2 Department of Linguistics, University of Hawai’i, Honolulu, 96822 USA

**Keywords:** Health care, Computational models

## Abstract

Dementia is a progressive neurological disorder that affects the daily lives of older adults, impacting their verbal communication and cognitive function. Early diagnosis is important to enhance the lifespan and quality of life for affected individuals. Despite its importance, diagnosing dementia is a complex process. Automated machine learning solutions involving multiple types of data have the potential to improve the process of automated dementia screening. In this study, we build deep learning models to classify dementia cases from controls using the Pitt Cookie Theft dataset from DementiaBank, a database of short participant responses to the structured task of describing a picture of a cookie theft. We fine-tune Wav2vec and Word2vec baseline models to make binary predictions of dementia from audio recordings and text transcripts, respectively. We conduct experiments with four versions of the dataset: (1) the original data, (2) the data with short sentences removed, (3) text-based augmentation of the original data, and (4) text-based augmentation of the data with short sentences removed. Our results indicate that synonym-based text data augmentation generally enhances the performance of models that incorporate the text modality. Without data augmentation, models using the text modality achieve around 60% accuracy and 70% AUROC scores, and with data augmentation, the models achieve around 80% accuracy and 90% AUROC scores. We do not observe significant improvements in performance with the addition of audio or timestamp information into the model. We include a qualitative error analysis of the sentences that are misclassified under each study condition. This study provides preliminary insights into the effects of both text-based data augmentation and multimodal deep learning for automated dementia classification.

## Introduction

Dementia is a complex disease associated with declines in cognitive functions such as memory, thinking, and reasoning. There exists an estimated 47.5 million people globally who are affected by dementia, with some portion demonstrating severe emotional and language impairments^1^.

The diagnostic process for dementia requires an overall review of the patient’s medical history, genetic testing, psychiatric evaluations, and cognitive assessments, often supplemented by neuroimaging techniques^2,3^. This multi-faceted nature of dementia diagnosis is complex, leading to growing interest in simplifying the process using more accessible and lower-cost methods^4–6^. Among the cognitive problems caused by dementia, verbal and speech impairments can be easily observed. Therefore, verbal fluency features can serve as a promising diagnostic biomarker^7^.

One widely used assessment for verbal fluency is to elicit participants’ responses to visual stimuli, measuring their ability to retrieve lexical items. DementiaBank^8^, the largest publicly available dataset related to dementia, provides such data collected from patients who underwent such assessments. DementiaBank includes audio recordings and text transcripts, serving as a useful resource for machine learning (ML) modeling of dementia.

Prior research efforts have built machine learning models for classifying dementia, with the end goal of creating a screening tool or diagnostic aid. Some studies fine-tuned pre-existing language models^9^, while others developed models from scratch^10^. Most prior work focused on singular data types—either audio^11,12^ or text data^13^—for model training. Only a few prior studies have explored the synergistic effects of integrating these data types into a single multimodal model ^14,15^.

In this study, we combined multiple data modalities—audio, text, and timestamps - from DementiaBank to classify dementia using short participant responses to the structured task of describing a picture of a cookie theft. We fine-tuned pre-trained Wav2vec and Word2vec models and tested them with a text-based data-augmentation method: synonym replacement.

The remainder of this paper is structured as follows: Section “Related work” is an overview of relevant literature; Section “Methods and models” provides details of our six experimental models, each crossed with different combinations of data modalities and the data augmentation method; Section “Results” includes our experimental results and discussions; Section “Discussion and conclusion” summarizes our observations and provides future directions for this type of research. To our knowledge, this is the first study to incorporate timestamps with text and audio data for a multimodal approach to automated dementia diagnosis.

## Related work

Previous studies have focused on detecting a specific type of dementia, such as Alzheimer’s Disease (AD). Within DementiaBank, the Alzheimer’s Dementia Recognition through Spontaneous Speech (ADReSS) Challenge^10^ contains multiple shared tasks, allowing researchers to base their methodologies on common datasets for comparative analysis. Prior AD classification techniques in these shared tasks have leveraged fine-tuning of existing models, data augmentation, and feature engineering. Studies that utilized feature engineering^10,12,14,16^ extracted audio and text features—either manually or through existing models—and trained models on a binary classification task.

Other studies have fine-tuned pre-trained language models like BERT^17^ and obtained high performances^13,16^. Data augmentation strategies including audio and text augmentation techniques (noise, lexical substitution, and paraphrasing) were also applied to handle the challenges associated with data sparsity^15^.

In addition to aiming for high performance in classification tasks, an important objective is to identify features that can assist with AD diagnosis in clinical settings. Some studies emphasized various semantic and lexico-syntactic features such as the proportion of personal pronouns and average sentence length^16^.

Beyond the ADReSS Challenge, researchers have also explored the Pitt Corpus^18^ within DementiaBank. Some studies constructed models from scratch^19^ while others leveraged pre-existing models^20^. Among these, some studies solely used text transcripts^21^, while others focused exclusively on audio recordings^22^. Only a few integrated multiple modalities, including both audio and text data^14,23,24^.

To summarize, it has been suggested in the existing literature that a multimodal approach that integrates different types of data, such as audio, text, and timestamps, can potentially lead to a more effective approach to the classification of dementia. Traditional methods often relied on a single data type, which may not capture the complexity of the condition. Although some studies reviewed above have used multiple modalities and suggested that embedding-based models can be promising^23,24^, further examination is needed to understand the synergistic performance of multiple embedding models, particularly across audio and text modalities. We hypothesized that the combination of Wav2vec and Word2Vec, two popular embeddings that have not yet been explored for dementia classification to the best of our knowledge, might classify dementia more effectively than using either feature extractor alone.

## Methods and models

We evaluated two data modalities, audio and text, as well as text-based synonym data augmentation and the inclusion of timestamps as a model input.

### Datasets and data preprocessing

#### Data source

We used the “Pitt Cookie Theft” dataset from DementiaBank^18^. This dataset contains participants’ responses when they were asked to describe what they saw in a stimulus photograph depicting a cookie theft. We selected this dataset because it contains timestamps for each word, allowing us to study the incorporation of an explicit time representation - analogous to positional embeddings in many large language models. It should be noted that the dataset also included a few non-AD patients, with conditions such as Parkinson’s and depression. We kept these datapoints because their relatively small proportion was not expected to affect the dataset’s overall representation of AD patients.

#### Data preparation

Because participant descriptions of of the cookie theft image tended to be brief, both audio and text data were divided into individual sentences, with each sentence being considered as a single datapoint. There were a total of 9447 such datapoints, of which 3873 were from dementia patients and 5574 from controls. The control datapoints were sentences spoken by investigators as well as those spoken by patients in the control data. Dementia datapoints, on the other hand, were sentences spoken by dementia patients.

In order to process audio data, the dataset was first processed through a Wav2vec feature extractor, with similar sampling rates used during the model’s pre-training. The text data underwent tokenization using the index token of a custom dictionary, enabling the mapping of words to their corresponding pre-trained word2vec embeddings in Gensim’s ‘word2vec-google-news-300’^25^. Words without corresponding embeddings were marked as Out-of-Vocabulary (OOV) and were represented by zero vectors.

We retained the starting times and ending times of each word. The timestamp for the first word in each sentence was normalized to start at 0 and processed as decimal digits.

Four dataset conditions were created:

*Original condition*: the original dataset with 9447 datapoints, including 3873 dementia and 5574 control datapoints.

*Shorts-removed condition*: excluded sentences shorter than two words, resulting in 4318 control and 3368 dementia datapoints.

*Original-augmented condition*: augmented from the dataset in the Original Condition, leading to 31,273 control and 22,664 dementia datapoints.

*Shorts-augmented condition*: augmented from the dataset in the Short-Removed Condition, yielding 28,964 control and 22,039 dementia datapoints.

For all four conditions, the datasets were randomly divided into training and test sets using a 4:1 ratio. Furthermore, the training datasets were also split into training and validation segments using a 4:1 ratio. These splits were used to perform fivefold cross-validation with hyperparameter optimization.

#### Ethics and inclusion statement

Ethical approval was obtained in writing from the DementiaBank (https://dementia.talkbank.org) owners to obtain access to the database. This database has specific ground rules in place, including fundamental data sharing rules, principles, and a code of Ethics of TalkBank designed to protect confidentiality (https://talkbank.org/share/rules.html). All authors have followed the ground rules in using the database for this research. The speech recordings used were handled in strict confidence.

Since our research did not require the collection of new data from live subjects with dementia during the model’s training or evaluation, the requirement for ethical approval by an ethics review committee was not required. We conducted our experiments solely with data from DementiaBank, which did not involve any recruitment from our end. Our methods were therefore in strict compliance with the appropriate standards and directives, such as the Declaration of Helsinki.

All individuals whose recordings were used from DementiaBank had provided their informed consent before the inclusion of their data into the database. We anticipate no legal, social, or financial implications arising from this study.

### Audio model

**Figure 1:**
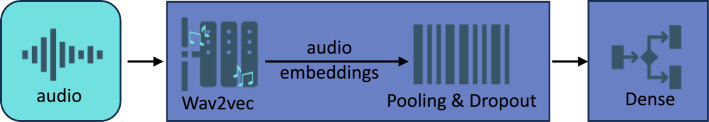
Architecture of the audio-only classification model.

We created an audio model (Fig. 1), which was fine-tuned using Wav2vec as the baseline representation. The audio data was processed through Wav2vec to obtain audio embeddings and was passed to a dense layer for binary classification. We used binary cross-entropy loss for optimization. We note that the weights from the pretrained Wav2vec feature extractor were frozen during the training and only the other layers of architecture were updated.

#### Wav2vec

Wav2vec^26^ is a self-supervised convolutional architecture that transforms audio waveforms into embeddings. Initially trained on unlabeled audio data, these embeddings were passed through a transformer for a masked prediction task. In this task, half of the audio embeddings were masked and predicted using the remaining unmasked portions. Wav2vec is particularly notable in speech recognition tasks due to its adaptability to various audio recordings and its superior performance compared to prior methods.

### Text model

**Figure 2:**
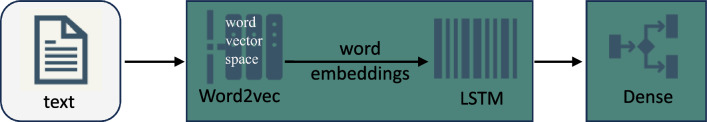
Architecture of the text-only classification model.

The text model (Fig. 2) included the embedding layers from Word2vec and used an LSTM model that was connected to a dense layer for final classification.

#### Word2vec

Word2vec^27^ is a feed-forward neural network that is designed to produce vector representations of words. It uses surrounding words as input to generate these vectors, and captures semantic relationships between the words. The generated vectors position semantically similar words closer in the vector space. As with the audio model, the weights from the pretrained Word2vec feature extractor were frozen during the training and only the other layers of the architecture were updated.

#### LSTM

We used an LSTM model with 16 units to process embedded sentences and used a recurrent dropout rate of 0.2. A dense layer with sigmoid activation was appended to the LSTM layer to perform binary classification.

### Timestamps

**Figure 3:**
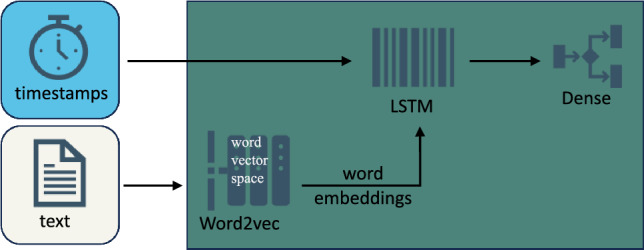
Architecture of the model combining text and timestamps.

**Figure 4:**
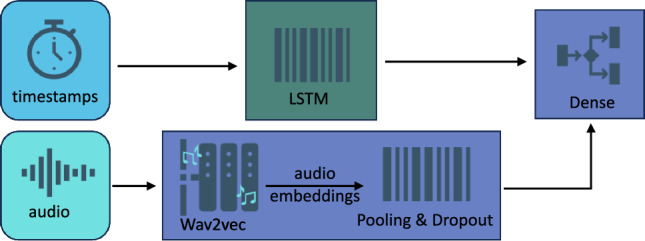
Architecture of the model combining audio and timestamps.

Timestamps for each word were extracted from the corpus. In the text + time model (Fig. 3), timestamps were concatenated with the word embeddings before feeding them as input into subsequent layers. In the audio + time model (Fig. 4), timestamps were passed through an LSTM layer first, and later were concatenated with the audio embeddings, which were passed through an average pooling layer. Finally, the concatenated output was passed through the dropout layer before final classification.

### Concatenated models

**Figure 5:**
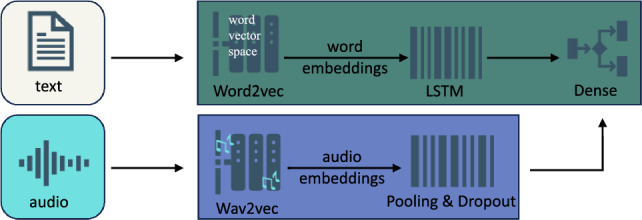
Architecture of the model combining audio and text.

**Figure 6:**
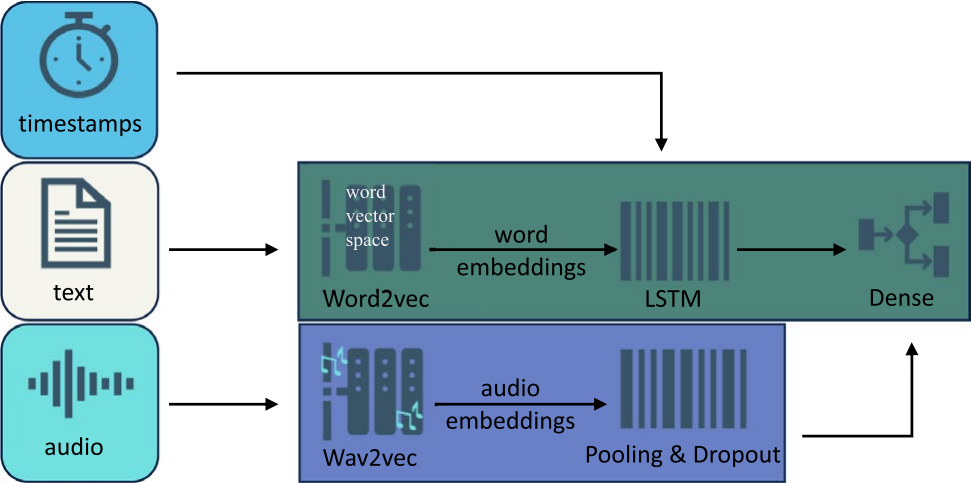
Architecture of the model combining all three modalities.

In the concatenated audio-text model (Fig. 5), word embeddings from the text model were processed through an LSTM layer. The audio model was then passed through the same average pooling and dropout layers before concatenation with the text model. A final dense layer was added for classification. We also developed a model combining data from audio, text, and timestamps (Fig. 6). The architecture for these models remained consistent with the previously described models. These segments were concatenated for the final classification task.

### Data augmentation

Due to the relatively small dataset size, we implemented text-based data augmentation. Specifically, we used the Synonym Replacement (SR) method^28,^ where a synonym for a word was used to create a duplicated sentence with the original word replaced by its synonym. Each word was replaced by its synonym once (n=2). For instance, if a sentence contained 5 words, all of which had synonyms available in the NLTK dictionary, five new sentences were generated, each having one original word replaced by a synonym.

### Experimental setup

All models were trained for 50 epochs with a batch size of 16. The objective was to minimize binary cross-entropy loss. To prevent overfitting, early stopping was added to stop training if the validation loss failed to decrease for 10 consecutive epochs. All code was developed using the TensorFlow Keras library^29^.

## Results

The experiments were conducted with five separate and independent train-test splits to ensure generalizability and reliability. We report the mean and standard deviation for all results. We include five evaluation metrics: accuracy, precision, recall, F1 score, and AUC ROC scores. The highest test scores for each metric are noted in bold.

**Figure 7:**
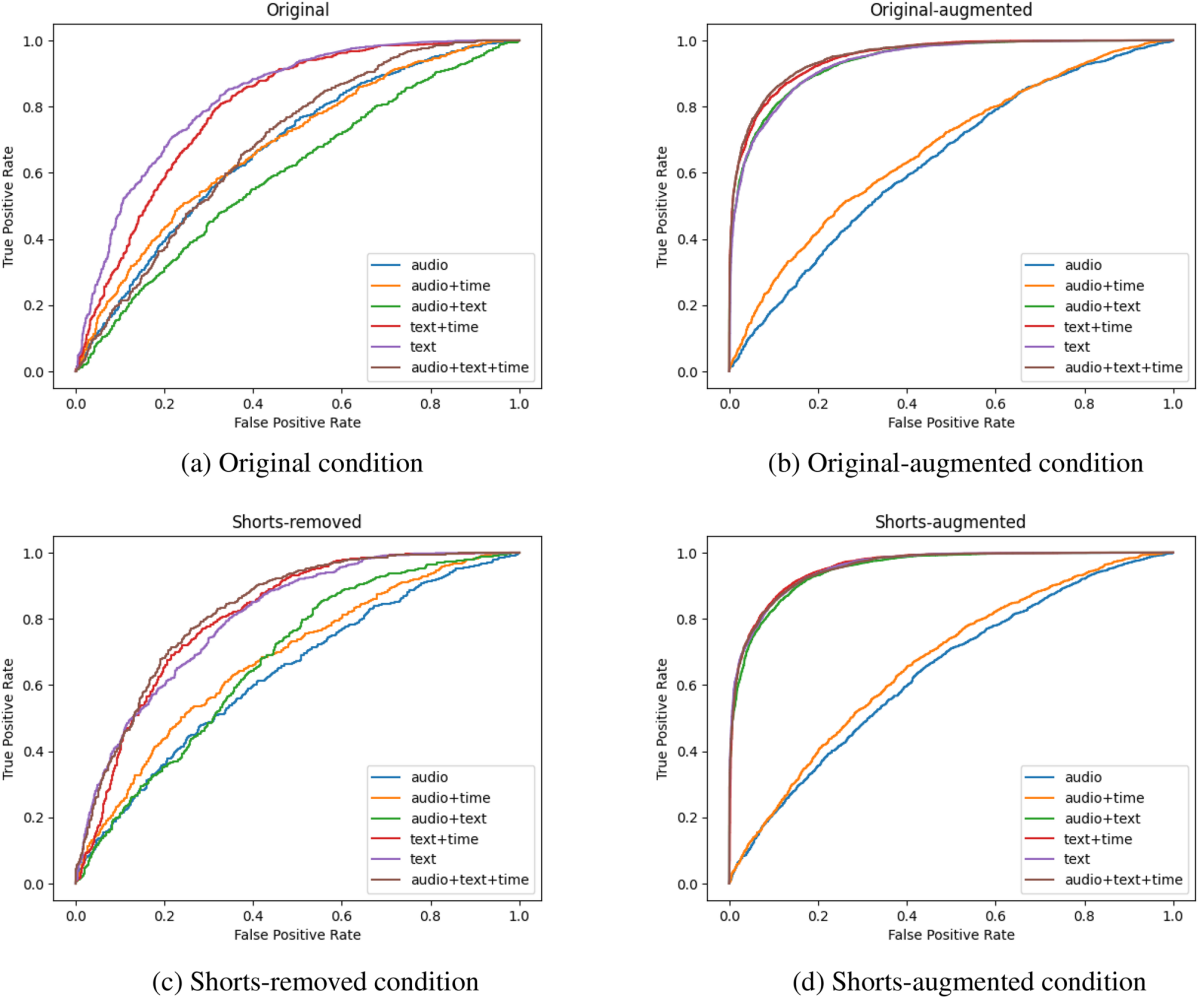
AUC-ROC curves for the four data augmentation conditions we evaluated: using original data, exclusion short sentences with the original data, augmenting the original data, and augmenting the data with the short sentences removed.

**Figure 8:**
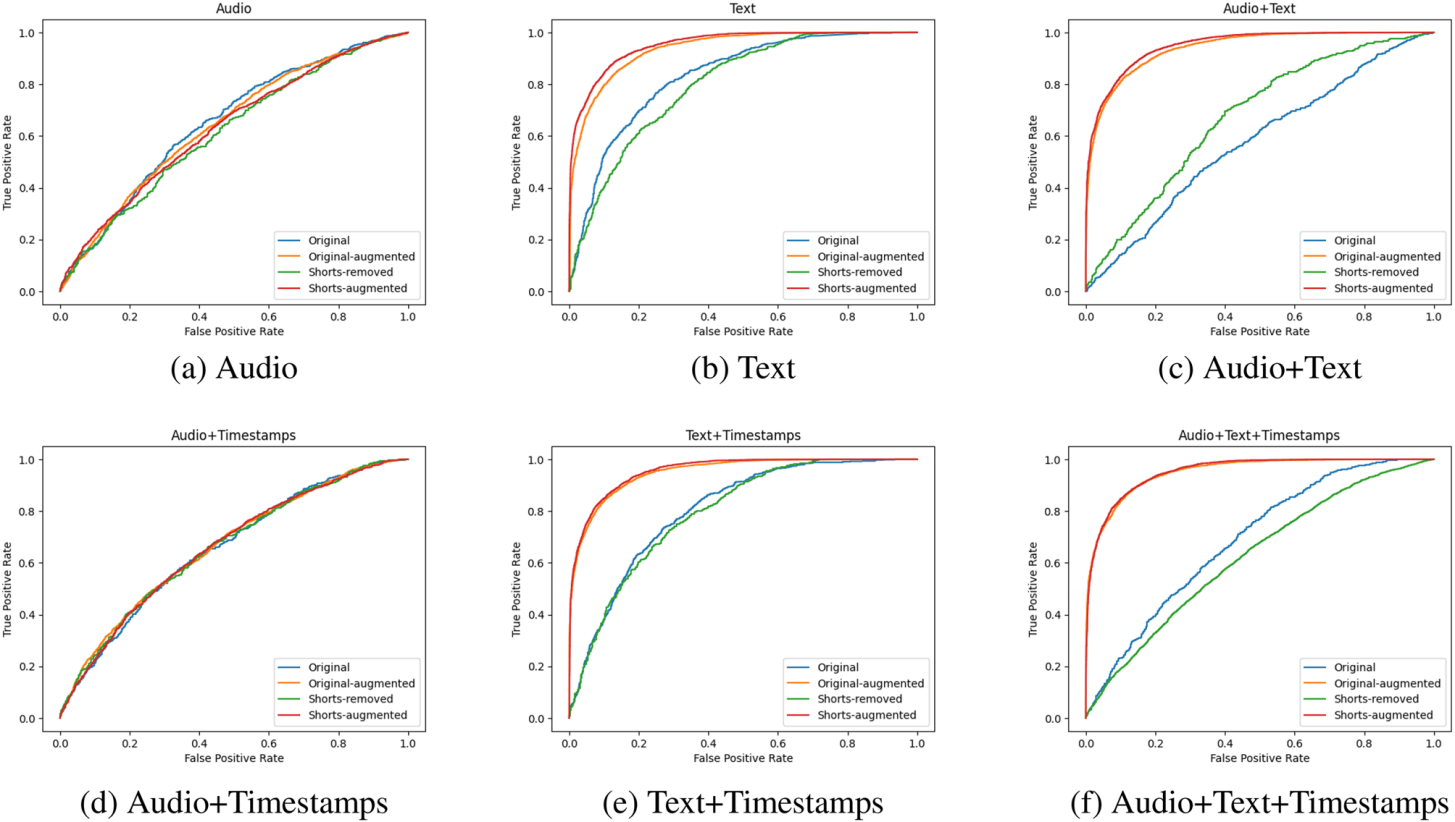
AUC-ROC curves for all six modality combinations we evaluated: audio only, text only, audio and text, audio and timestamps, text and timestamps, and all three.

Our results highlight the challenges and opportunities associated with multimodal dementia classification using speech data. As evidenced in Table 1 as well as Figs. 7a and 8a, unimodal audio models underperformed compared to the text models. The audio+time model (Fig. 8d) also yielded suboptimal results. This suggests that the audio modality may be challenging to engineer with current state-of-the-art models such as Wav2vec. On the other hand, the text model (Table 1 as well as Figs. 7a and 8b) performed well, and its performance was even better when combined with time, as demonstrated by the superior performance of the text+time model (Table 1 and Figs. 7a, 8e,f).

**Table 1:** Results using the original data.

Original	Accuracy	Precision	Recall	F1-score	AUROC
Audio	0.6484 ± 0.008	0.593 ± 0.019	0.4425 ± 0.063	0.5039 ± 0.032	0.7085 ± 0.011
Text	**0.691** ± **0.034**	0.6484 ± 0.07	0.6299 ± 0.127	0.62765 ± 0.027	0.7638 ± 0.046
Audio $+$ Time	0.6123 ± 0.006	0.5565 ± 0.022	0.3286 ± 0.039	0.4115 ± 0.026	0.6517 ± 0.011
Text $+$ Time	0.6909 ± 0.013	**0.6537** ± **0.036**	0.5566 ± 0.4463	0.5995 ± 0.022	**0.7647** ± **0.018**
Audio $+$ Text	0.6731 ± 0.04	0.5958 ± 0.047	**0.6852** ± **0.097**	**0.6341** ± **0.048**	0.7448 ± 0.045
Audio $+$ Text $+$ Time	0.6539 ± 0.031	0.5874 ± 0.07	0.5301 ± 0.138	0.55 ± 0.087	0.7161 ± 0.043

Results of the best-performing modality for each metric are in bold.

We observe higher standard deviations in some modalities, mostly in the audio-based models, suggesting that the model was more prone to poor fitting in several data splits. This is likely due to the failure of the audio embedding to yield a good classification signal.

As observed in Table 2 and Fig. 7c (the audio+text+time model we saved had an above-average performance), the exclusion of shorter sentences during preprocessing did not lead to significant improvement in the overall model performance. However, Table 3, Fig. 7b, Table 4 and Fig. 7d show a noticeable improvement after data augmentation was applied. AUROC scores in models using text data surpassed 90% (Fig. 8b–e), and both accuracy and F1 scores were consistently above 80%. This uplift in performance suggests that the text-based augmentations based on synonym replacement may have captured important semantic features related to dementia.

**Table 2:** Results using the original data with short sentences removed.

Shorts-removed	Accuracy	Precision	Recall	F1-score	AUROC
Audio	0.5958 ± 0.014	0.587 ± 0.046	0.2945 ± 0.108	0.3811 ± 0.084	0.624 ± 0.022
Text	**0.6861** ± **0.027**	**0.623** ± **0.042**	0.6951 ± 0.058	**0.6545** ± **0.022**	**0.7593** ± **0.024**
Audio $+$ Time	0.5897 ± 0.009	0.5631 ± 0.034	0.3554 ± 0.07	0.4306 ± 0.054	0.6163 ± 0.01
Text $+$ Time	0.6683 ± 0.029	0.6197 ± 0.043	**0.7011** ± **0.137**	0.6494 ± 0.032	0.7353 ± 0.047
Audio $+$ Text	0.6052 ± 0.014	0.5669 ± 0.024	0.5255 ± 0.152	0.534 ± 0.082	0.6482 ± 0.014
Audio $+$ Text $+$ Time	0.6257 ± 0.065	0.5724 ± 0.075	0.5239 ± 0.151	0.5415 ± 0.107	0.6769 ± 0.081

Results of the best-performing modality for each metric are in bold.

**Table 3:** Results using the augmented versions of the original data.

Original-augmented	Accuracy	Precision	Recall	F1-score	AUROC
Audio	0.6038 ± 0.036	0.5846 ± 0.021	0.1831 ± 0.04	0.2764 ± 0.044	0.6336 ± 0.003
Text	0.8294 ± 0.005	**0.8339** ± **0.022**	0.751 ± 0.042	0.7892 ± 0.013	0.9208 ± 0.002
Audio $+$ Time	0.6306 ± 0.068	0.5994 ± 0.012	0.3673 ± 0.044	0.4542 ± 0.033	0.6759 ± 0.008
Text $+$ Time	**0.8344** ± **0.006**	0.8267 ± 0.035	0.7721 ± 0.043	**0.797** ± **0.009**	**0.9236** ± **0.005**
Audio $+$ Text	0.825 ± 0.013	0.7978 ± 0.039	**0.7859** ± **0.056**	0.7899 ± 0.019	0.9124 ± 0.015
Audio $+$ Text $+$ Time	0.8315 ± 0.014	0.8212 ± 0.034	0.767 ± 0.012	0.7927 ± 0.014	0.9177 ± 0.014

Results of the best-performing modality for each metric are in bold.

**Table 4:** Results using the augmented versions of the data with short sentences removed.

Shorts-augmented	Accuracy	Precision	Recall	F1-score	AUROC
Audio	0.5954 ± 0.001	0.5989 ± 0.025	0.1939 ± 0.029	0.2914 ± 0.03	0.6258 ± 0.008
Text	0.841 ± 0.01	0.8212 ± 0.014	**0.8089** ± **0.024**	0.8148 ± 0.014	0.9276 ± 0.009
Audio $+$ Time	0.6254 ± 0.005	0.5894 ± 0.023	0.4298 ± 0.05	0.4951 ± 0.032	0.6692 ± 0.008
Text $+$ Time	**0.8478** ± **0.003**	**0.8375** ± **0.02**	0.8039 ± 0.207	**0.8199** ± **0.007**	**0.9345** ± **0.005**
Audio $+$ Text	0.835 ± 0.02	0.8154 ± 0.036	0.7982 ± 0.039	0.80591 ± 0.023	0.9216 ± 0.02
Audio $+$ Text $+$ Time	0.8451 ± 0.003	0.83646 ± 0.027	0.7992 ± 0.028	0.8166 ± 0.04	0.931 ± 0.005

Results of the best-performing modality for each metric are in bold.

### Qualitative error analysis

We conducted a qualitative error analysis to understand which types of prompt responses were frequently misclassified, providing insights into the types of sentences that may be archetypal of dementia. We observe the following patterns:

**False positives:** Our text model tended to misclassify certain types of sentences from control patients as dementia patients, providing insights into the types of prompt responses that patients with dementia may have commonly spoken. These sentences generally had one or more of the following characteristics:

- *Noun-phrase sentences*: Examples include ‘curtain on the window’, ‘down on this side of the picture’.
- *Ungrammatical sentences*: Sentence types that were uttered by patients in the control group but were slightly unnatural. Examples include ‘the boy is uh taking cookies out of the cookie jar’, ‘uh mother’s drying dishes’, and ‘that’s real good then’.
- *Repetition*: The repetition of patients’ sentences from the investigator, e.g., ‘climbing a stool’.

**False negatives:** Sentence archetypes from dementia patients that were misclassified as coming from control participants, providing insights into the types of prompt responses that the model learned are not specifically associated with dementia, include:

- *Correct and transcribed correctly:* Sentences that were grammatically correct and transcribed correctly. Examples include ‘that’s about all’, and ‘and the girl’.
- *Short and Correct:* Examples include sentences like ‘here,’ ‘okay’.
- *Common responses:* Sentences that patients often responded to or asked and were transcribed correctly ‘okay’, ‘that’s terrible’, ‘that’s about it, right?’

The original-augmented model often misclassified the following sentence archetypes:

- *Unlikely connotations*: Augmented sentences sometimes yielded unlikely or misleading connotations.
  - $\checkmark$ ‘I’ve got the tape recorder on so’.
    (original, control, predicted as control)
  - $\checkmark$ I’ve got the videotape recorder on so’.
    (augmented, control, predicted as dementia)
  - $\checkmark$ I’ve got the tape registrar on so’.
    (augmented, control, predicted as dementia)
- *Word usage*: Augmented words were common in control data and were sometimes present in sentences from dementia patients.
  - $\checkmark$ It shows the mother in the kitchen wiping dishes’.
    (original, dementia, predicted as dementia)
  - $\checkmark$ It **testify** the mother in the kitchen wiping dishes’. (augmented, dementia, predicted as control)
- *Augmented and incorrect sentences*: Sentences that were originally grammatical but became ungrammatical after augmentation. For example:
  - $\checkmark$‘The little girl’s standing there’.
    (original, dementia, predicted as dementia)
  - $\checkmark$ The little miss standing there’.
    (augmented, dementia, predicted as control)

In the Shorts-removed condition, the incorrectly predicted sentences generally were similar to that of the original dataset, minus the influence of short sentences. This suggests that the presence or absence of short sentences in the data did not dramatically affect the types of errors the model makes, implying that the model’s predictive ability is not significantly affected by sentence length alone. Interestingly, the errors made by the model in the Shorts-augmented condition were similar to those in the original-augmented condition. This might suggest the robustness of data augmentation, regardless of the presence or absence of short sentences. The findings further suggest that while data augmentation significantly enhanced the model’s overall performance, it did not necessarily change the nature of the mistakes made by the model in prediction.

## Discussion and conclusion

We have explored dementia classification by leveraging audio, text, and timestamp data from short participant descriptions of a visual stimulus. Using pre-trained models such as Wav2vec and Word2vec, we observed that the presence of text data seemed to bolster the performance of the model significantly, even making up for the more noisy and lower-performing audio data representation. This suggests that text-based data can be a crucial component for improving the diagnostic performance of dementia classification models applied to data collected in response to a prompt.

While the performance of audio and timestamp data was relatively modest, their inclusion within a multimodal framework did sometimes lead to marginal improvements. Further work is required to discover more successful ways to incorporate audio data into classification procedures. In particular, our results suggest that Wav2vec audio representations are insufficient for dementia classification in this context. This result is somewhat surprising in light of previous work that was able to classify autism using audio from naturalistic yet semi-structured home videos with Wav2vec feature representations^30^. Part of the success of these prior efforts is likely attributable to the relatively structured nature of the input audio, where fine-grained structure was imposed by the mechanics of a mobile game^31–35^. While the Cookie Theft task was structured in that the same visual stimulus was provided to all participants, we hypothesize that using a series of fixed specific questions about the content of the image rather than a single broad prompt could possibly improve prediction outcomes. Future work is required to properly extract audio features that are relevant to the classification of dementia using the DementiaBank data.

The limitations of our procedures are as follows. First, we only used a single dataset consisting of short responses to a very specific prompt, undermining the generalizability of this approach to other data collection procedures. Second, there might exist other audio representations that can enhance the overall performance, possibly leading to better performance of the audio modality. However, we only tried Wav2vec; further audio representations should be empirically examined. Third, we only integrated different modalities using basic concatenation, but other methods of multimodal fusion and an empirical study of the differences in performance between early and late-stage fusion architectures would lead to greater understanding of multimodality.

## Data Availability

The Pitt datasets used in this study are sourced from DementiaBank (https://dementia.talkbank.org), which requires approval for access. The datasets generated in the current study are available from the corresponding author upon reasonable request.

## Code availability

Code available at https://github.com/limkhaiin/dementia.
